# Rottlerin-Liposome Inhibits the Endocytosis of Feline Coronavirus Infection

**DOI:** 10.3390/vetsci10060380

**Published:** 2023-05-30

**Authors:** Jong-Chul Choi, Sung-Won Jung, In-Yeong Choi, Yeong-Lim Kang, Dong-Hun Lee, Sang-Won Lee, Seung-Yong Park, Chang-Seon Song, In-Soo Choi, Joong-Bok Lee, Changin Oh

**Affiliations:** 1 Qvet Co., Ltd., 606, Alumini Association Building of Konkuk University, 5 Achasan-ro 36-gil, Gwangjin-gu, Seoul 05066, Republic of Korea; mfilia@konkuk.ac.kr; 2 Laboratory of Infectious Diseases, College of Veterinary Medicine, Konkuk University, 120 Neungdong-ro, Gwangjin-gu, Seoul 05029, Republic of Korea; tjddnjs0204@naver.com (S.-W.J.); joealpha@naver.com (I.-Y.C.); vinlovehole@naver.com (Y.-L.K.); donghunlee@konkuk.ac.kr (D.-H.L.); odssey@konkuk.ac.kr (S.-W.L.); paseyo@konkuk.ac.kr (S.-Y.P.);; 3 KU Research Center for Zoonosis, 120 Neungdong-ro, Gwangjin-gu, Seoul 05029, Republic of Korea; 4 Department of Genetics, Yale School of Medicine, P.O. Box 208005, New Haven, CT 06520-8005, USA

**Keywords:** feline infectious peritonitis, rottlerin, drug delivery system, liposome, antivirals

## Abstract

**Simple Summary:**

Rottlerin, a type of natural extract, can inhibit the activity of various viruses. Feline coronavirus (FCoV) causes a devastating disease in cats. In this study, we investigated whether rottlerin has inhibitory effects on FCoV. We demonstrated that rottlerin inhibits FCoV replication and FCoV-induced PKCδ phosphorylation. We showed that rottlerin affects the early (endocytosis) and late (syncytium formation) stages of FCoV replication. We also observed that the liposome encapsulation technique overcomes the drawbacks of rottlerin and enhances its efficacy. Therefore, we suggest that rottlerin-liposome is worth further investigation as a potential treatment for FCoV.

**Abstract:**

Rottlerin (R) is a natural extract from *Mallotus philippensis* with antiviral properties. Feline infectious peritonitis (FIP) is a fatal disease caused by feline coronavirus (FCoV) that is characterized by systemic granulomatous inflammation and high mortality. We investigated the antiviral effect of liposome-loaded R, i.e., rottlerin-liposome (RL), against FCoV. We demonstrated that RL inhibited FCoV replication in a dose-dependent manner, not only in the early endocytosis stage but also in the late stage of replication. RL resolved the low solubility issue of rottlerin and improved its inhibition efficacy at the cellular level. Based on these findings, we suggest that RL is worth further investigation as a potential treatment for FCoV.

## 1. Introduction

Rottlerin (R) is a natural polyphenol ketone compound derived from the fruit powder of *Mallotus philippensis*, commonly known as the Kamala tree. A traditional Indian medical journal, *Ayurveda*, has referred to the powder as *Kampillaka* and recommended its use as a remedy for topical and oral medicine in the treatment of various diseases since the 8th century [1,2,3,4]. When applied externally, *Kampillaka* has been known to alleviate skin disease and injuries and was especially used to treat carbuncles in combination with cow’s ghee to form an ointment [4]. Internally, *Kampillaka* has been used to treat ascites, abdominal tumors, parasite infections, blood disorders, contraception, and leprosy [4]. The recommended dosage of *Kampillaka* for children is 500 mg to 750 mg and for adults is 8 g [4]. R is also known to possess several medicinal properties, including anticancer, anti-inflammatory, antioxidant, and hepatoprotective effects [5,6]. Intriguingly, several studies have demonstrated that R exhibits inhibitory activity against various pathogens, including parasites, bacteria, and viruses [6,7,8,9,10,11,12,13].

R has been widely used as a protein kinase C δ (PKCδ) phosphorylation inhibitor [10,12,14]. Research has shown that rottlerin blocks the phosphorylation of PKCδ as a secondary change, acting as a mitochondrial uncoupler [15]. However, previous studies showed that some viruses (human immunodeficiency virus 1 and porcine reproductive and respiratory syndrome virus) are inhibited in a PKCδ-dependent pathway, while other viruses (e.g., rabies virus) are not [10,12,16]. In addition, there are studies showing that levels of PKCδ are increased during certain inflammation conditions, such as macrophage activation including LPS accumulation, *Mycobacterium tuberculosis* infection, and fibrosis [17,18,19]. Although not a specific PKCδ inhibitor, it is known that rottlerin can inhibit macrophages’ release of inflammatory factors [18].

Drug delivery systems (DDSs) have been widely used in various fields to increase the efficacy of pharmaceutical substances [20,21,22]. Among them, liposomes have advantages in improving the solubility, delivery efficiency, and storage stability of substances [23,24,25]. Liposome technology has been applied to natural extracts to address various issues, including their highly hygroscopic structure, low solubility, poor bioavailability, instability, and high dose-induced toxicity [25,26]. Good examples of liposome natural extracts being applied to pharmaceutical substances are curcumin, fat-soluble vitamins, and polyphenols from tea [27,28,29].

Feline coronavirus (FCoV; order: *Nidovirales*; subfamily: *Coronavirinae*; family: *Coronaviridae*) is a known causative agent of a devastating disease, feline infectious peritonitis (FIP), which is characterized by systemic granulomatous inflammation [30,31]. Clinically, FIP can be categorized into wet, dry, and mixed forms [32]. All forms cause granulomatous lesions consisting of macrophages and other inflammatory cells in multiple organs, such as the omentum, mesenteric lymph nodes, spleen, and liver [33]. Clinical signs include fever, jaundice, abdominal effusions, and weight loss, and may also affect the eyes and central nervous system [32,34]. The average survival time was reported to be 21.3 days for the wet form, 38.4 days for the dry form, and 110.9 days for the mixed-type form [35]. Even in cases where apparent recovery has occurred, signs may recur several months to several years later [36]. 

Controlling and treating infections due to FCoV is challenging due to its epidemiological characteristics. FCoV primarily infects kittens during the nursing period and causes mild enteric disease [30,37]. Therefore, early management, such as separating young cats from their mother, is necessary to prevent the infection and development of FIP [37,38]. There are several antiviral candidates for FCoV, including GS-441524, Mefloquine, Molnupiravir, Ribavirin, GC376, U18666A, and Itraconazole, which have undergone animal or clinical trials [34,39,40,41,42,43,44,45]. 

Previously, our group demonstrated that rottlerin-liposome (RL), i.e., liposome-encapsulated rottlerin, successfully inhibited porcine reproductive and respiratory syndrome virus (PRRSV) infection in MARC-145 cells and piglets [12]. In detail, RL was able to inhibit PRRSV replication in cell cultures by up to −3 log (50% tissue culture infectious dose (TCID_50_)/mL) in a PKCδ-dependent manner. In animal studies, intranasal administration of RL significantly reduced viremia levels, interstitial pneumonia, and clinical scores of PRRSV-infected piglets compared to the control group. 

Thus, we hypothesized that FCoV, which belongs to the order *Nidovirales*, along with PRRSV, may also be inhibited by RL. In this study, we confirmed that RL can inhibit the replication of FcoV. We can observe a slight reduction in PKCδ phosphorylation by RL treatment during the early stages of FCoV infection. RL affects at least two stages of FCoV replication, one during the early endocytosis of FCoV and the other during the late stage, which is possibly related to the syncytia formation stage. Our study provides preliminary evidence of the inhibitory effect of RL on FCoV and its possible mechanism and highlights the potential advantages of applying liposomes to antiviral drugs.

## 2. Materials and Methods

### 2.1. Cells, Virus, and Antibodies

In this study, we used the WSU 79-1146 (VR-990) virus as a model for FCoV [46]. Crandell feline kidney (CRFK) cells were grown according to ATCC handling guidelines for the virus. The cells were maintained using Eagle’s Minimum Essential Medium (EMEM) with 10% fetal bovine serum (FBS) (Thermo Fisher Scientific, Cleveland, OH, USA) and 1% Antibiotic-Antimycotic solution (100×) (Thermo Fisher Scientific, Cleveland, OH, USA), and the concentration of FBS was changed to 2% for virus propagation. For the immunofluorescence assay and Western blot, mouse anti-FCoV nucleocapsid (N) IgG, clone FIPV3-70 (BioRad, Hercules, CA, USA), PKCδ (D10E2) rabbit mAb (Cell Signaling Technology, Danvers, MA, USA), anti-PKC delta (phospho S645) antibody (Abcam, Cambridge, MA, USA), horseradish peroxidase (HRP)-conjugated goat anti-rabbit IgG (H+L) (Thermo Fisher Scientific, Cleveland, OH, USA), HRP-conjugated goat anti-mouse IgG (H+L), Alexa Fluor 488 (Thermo Fisher Scientific, Cleveland, OH, USA), or HRP-conjugated goat anti-mouse IgG (Abcam, Cambridge, MA, USA) was used [12,47,48]. Anti β-actin antibody, HRP (Abcam, Cambridge, MA, USA) was used for β-actin staining in the Western blot. All antibodies used in the Western blot were diluted with phosphate-buffered saline (PBS) containing 5% bovine serum albumin (BSA) (Sigma-Aldrich Chemie GmbH, Steinheim, Germany) and 0.05% Tween20 to block non-specific binding. We used a cell culture medium containing dimethyl sulfoxide (DMSO) (Thermo Fisher Scientific, Cleveland, OH, USA) as a control. 

### 2.2. Preparation of R and RL

R (Santa Cruz Biotechnology, Dallas, TX, USA) was dissolved in DMSO at a concentration of 10 mg/mL. RL was prepared using the thin-film hydration method with reference to previous studies [12,24]. Hydrogenated soy phosphatidylcholine (HSPC) (Avanti Polar Lipids, Alabaster, AL, USA) and cholesterol (Avanti Polar Lipids, Alabaster, AL, USA) in a ratio of 55:45 (mol/mol) were added to a flask containing ethyl acetate (Sigma-Aldrich Chemie GmbH, Steinheim, Germany) (1:2 *v*/*v*). Then, R was added at 1/20 of the weight of the mixture. The mixture was dissolved by stirring at room temperature for 1 h, the flask was mounted onto a rotary evaporator (WIGGENS, Straubenhardt, Germany), and the device was operated under conditions of 54 °C, 50 rpm, and 300 mbar to form a thin layer of film on the surface of the flask. Distilled water (DW) was added to hydrate the layer at 65 °C and 60 rpm. After the film was fully hydrated, the recovered product was dialyzed against DW overnight at room temperature.

### 2.3. Characterization of RL

To verify the quality of the RL, we measured its shape, size, and concentration. The shape of the particles was evaluated by utilizing a transmission electron microscope (TEM), while the size distribution was determined using a dynamic light scattering (DLS) system (Wyatt Technology, Santa Barbara, CA, USA). To determine the amount of R encapsulated in liposome, triton-x100 was mixed with the prepared RL at a ratio of 19:1 to dissolve the lipid (*v*/*v*), and the absorbance was measured at a wavelength of 405 nm using a spectrophotometer (TECAN, Männedorf, Switzerland) [49]. The absorbance values obtained were converted to concentrations using a standard curve.

### 2.4. Cell Viability Assay

Twenty-four hours prior to the test, CRFK cells were seeded at a concentration of 2 × 10^5^ cells/mL in 96-well plates. Various concentrations of R and RL were prepared by diluting them with a cell culture medium, and 100 µL of each concentration was added to the wells. DMSO was added as a control. The plates were then incubated at 37 °C in 5% CO_2_ for 48 h. Following this, 10 µL of EZ-Cytox (water-soluble tetrazolium salts) solution (Daeil Lab Service, Seoul, Korea) was added to each well and further incubated for 4 h [12]. At the end of the incubation period, the optical density of the wells was measured at a wavelength of 450 nm using a spectrophotometer (TECAN, Männedorf, Switzerland). The optical density value of the control group was considered to be 100% and each value was normalized accordingly.

### 2.5. Dose-Dependent Inhibition Assay

The CRFK cells were seeded at a concentration of 10^5^ cells per well in 24-well plates. The following day, we tested the anti-FCoV activity of various concentrations of R or RL, which was determined based on the cell viability assay results. 

The cells were pre-treated with various concentrations of R, RL, or DMSO (as vehicle controls) for 2 h before infection with FCoV at a multiplicity of infection (MOI) of 0.008 with the same drug concentrations. The cytopathic effect (CPE) induced by the virus was monitored for up to 72 h. Following this, the supernatant of the cell lysates was collected and subjected to titration or RT-qPCR.

### 2.6. RNA Extraction and RT-qPCR Assay

Viral RNA was isolated using a Viral Gene-spin™ Viral DNA/RNA Extraction Kit (iNtRON, Gyeonggi-do, Korea). To quantify viral RNA, we used the primers and probe set, targeting the 3’UTR region of FCoV from the previous study by modifying the reporter fluorescence to FAM [34]. The reaction mixes were prepared using the RNA UltraSense™ One-Step Quantitative RT-PCR System (Thermo Fisher Scientific, Cleveland, OH, USA) according to the manufacturer’s guidelines. The prepared mixture was incubated at 50 °C for 15 min and at 95 °C for 2 min, followed by 40 repetitions of the reaction at 95 °C for 15 s and 60 °C for 30 s using the LightCycler^®^ 96 Real-Time PCR System (Roche, Basel, Switzerland). The obtained quantification cycle (Cq) value was converted into a log (TCID_50_/mL) equivalent using a standard curve.

### 2.7. Western Blot

The CRFK cells (2 × 10^5^ cells/mL) were prepared in a 24-well plate and incubated for 24 h. After removing the medium, a medium containing 1 MOI of FCoV and 2.5 μM of RL was added. After 15, 30, 45, and 60 min, the cell lysates were collected using M-Per^®^ Mammalian Protein Extraction Reagent (Thermo Fisher Scientific, Cleveland, OH, USA), and all cell debris was removed by centrifugation at 13,000 rpm for 10 min at 4 °C. 

For sodium dodecyl-sulfate polyacrylamide gel electrophoresis (SDS-PAGE), samples were processed with a 4× LDS sample buffer (Thermo Fisher Scientific, Cleveland, OH, USA) according to the manufacturer’s guidelines. An amount of 10 μL of the prepared sample was loaded onto Bolt™ 4 to 12%, Bis-Tris gel, and electrophoresed at 200 volts for 25 min. Then, the bands were transferred to a nitrocellulose membrane under the condition of 20 volts for 1 h, as suggested by the manufacturer. The transferred membrane was washed 3 times with the washing buffer (PBS containing 0.05% Tween20) and stained with the primary antibody diluted with a blocking buffer (PBS containing 0.05% Tween20 and 5% BSA). After overnight incubation at 4°C, the membrane was washed 3 times and incubated with the secondary antibody diluted with a blocking buffer. After incubation for 2 h, the membrane was washed 3 times with PBS and developed using the Clarity Western ECL Substrate (Bio-Rad, Hercules, CA, USA). Images were taken using the FUSION SOLO system (Vilber Lourmat, Marne-la-Vallée, France). For the β-actin signal, the previously stained membrane was stripped with the Restore™ Stripping Buffer (Thermo Fisher Scientific, Cleveland, OH, USA) for 15 min and then stained with Anti β-actin antibody, HRP (Abcam, Cambridge, MA, USA) for 2 h and analyzed. The captured images were quantified using ImageJ [50]. Values of phosphorylated PKCδ (P-PKCδ) were normalized to those of total PKCδ (T-PKCδ). The relative P-PKCδ was calculated as a fold change of the normalized values to the negative control (NC, FCoV−/RL−) of each time point.

### 2.8. Time of Drug Addition Assay

Based on the virus inoculation time point (+0 h), 7 groups (A to G) of the inoculation times of the RL were created. Twenty-four hours before the inoculation, the 2 × 10^5^ cells/mL CRFK cells were prepared on a 24-well plate. At a predetermined point, the medium in each well was removed and 2.5 μM of the RL was added. At +0 h, a medium containing 0.008 MOI of FCoV and 2.5 μM of RL was added, incubated for an hour, and removed. The cells were further incubated for up to 72 h with the media containing 2.5 μM of the RL and the cell lysates were collected. The samples were subjected to titration or qRT-PCR. 

### 2.9. Immunofluorescent Assay

The CRFK cells were prepared at 2 × 10^5^ cells/mL in a 24-well plate and cultured for 24 h. After removing the medium, a medium containing 0.008 MOI of FCoV and 2.5 μM of R or RL was added. After 1 h, the medium was removed, and the cells were washed with PBS. After that, a medium containing 2.5 μM of R or RL was added and cultured for 24 h. After treatment, the cells were washed once with PBS and then fixed with 4% paraformaldehyde for 15 min, followed by permeabilizing for 15 min using 0.05% triton-X100. After washing 3 times with PBS, the primary antibody diluted with a blocking buffer was added to the cells. After overnight incubation at 4 °C, the cells were washed 3 times with a washing buffer, and the diluted secondary antibody was added. After incubation for 3 h, the cells were washed 3 times and mounted. The cells were observed using EVOS^®^ FL Color (Thermo Fisher Scientific, Cleveland, OH, USA). The captured images were quantified using ImageJ [50].

### 2.10. Internalization Assay

The CRFK cells were seeded on a chamber slide at a concentration of 1 × 10^5^ cells/mL and incubated for 24 h. The cells were pre-treated with the RL at a concentration of 2.5 μM and incubated for 1 h. After discarding the medium, the cells were inoculated with a mixture of 1 MOI of FCoV and 2.5 μM of RL. After 1 h of incubation for viral binding and internalization, the cells were washed and replenished with a medium containing 2.5 μM of RL. In the mock test group, a fresh medium containing DMSO was used. After 2, 4, and 6 h, each well was treated with PBS either containing or not containing protein kinase (Takara Korea Biomedical Inc., Seoul, Korea) for 45 min at 4 °C, then fixed, stained, and observed under an LSM-900 (Carl Zeiss, Göttingen, Germany). Microscopic images were processed using ImageJ software and fluorescence signals were quantified [50]. The fluorescence intensity from at least 50 cells for each group was averaged. 

### 2.11. Statistical Analysis

All statistical analyses were conducted using GraphPad Prism version 8 (GraphPad Software, Inc., San Diego, CA, USA). The 50% cytotoxicity concentrations (CC_50_) and 50% effective concentrations (EC_50_) were calculated using nonlinear regression analysis. Depending on the variability of the data, either one-way ANOVA and Dunnett’s multiple comparison or two-way ANOVA and Tukey’s multiple comparison were applied. Statistical significance is indicated by the symbols (*), (**), (***), and (****), representing *p* < 0.05, *p* < 0.005, *p* < 0.0005, and *p* < 0.0001, respectively.

## 3. Results

### 3.1. RL Inhibits FCoV Replication

**Figure 1:**
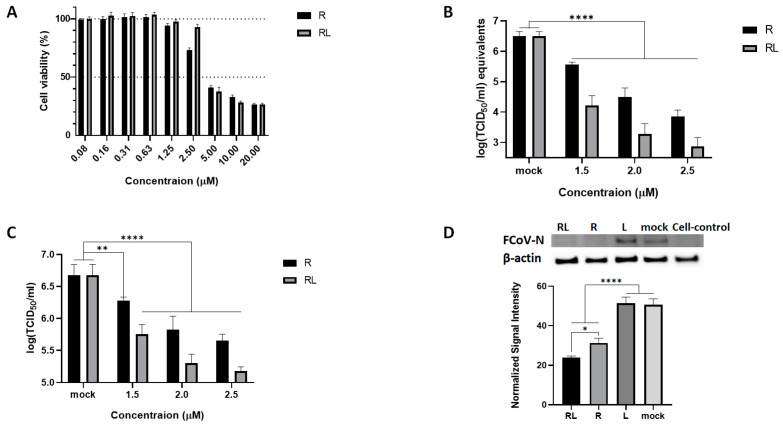
RL inhibited FCoV replication in a dose-dependent manner. (**A**) Confluent CRFK cells were treated with R and RL at the indicated concentrations and incubated for 48 h. After that, cell viability was measured using a water-soluble tetrazolium salts (WST) assay. (**B**,**C**) Pre-treated (indicated concentrations of R or RL) CRFK cells were infected with FCoV (MOI = 0.008) with R or RL for 1 h, followed by the same R or RL treatment for up to 72 hpi. The amount of virus was measured using RT-qPCR (**B**) and titration (**C**). (**D**) Confluent CRFK cells were infected with FCoV (MOI = 0.008) with R (2.5 μM), RL (2.5 μM), or liposome. At 24 h post-infection, cell lysates were examined using Western blot and the intensity of the bands was quantified using Image J and normalized to the value of β-actin. Statistical differences were analyzed using one-way ANOVA and Dunnett’s multiple comparison tests. The symbols (****), (**), and (*) indicate *p* < 0.0001, *p* < 0.005, and *p* < 0.05, respectively (please find the WB full membrane in Figure S3).

To investigate the effect of RL on the replication of FCoV in the cell culture, we first prepared RL by loading R into a lipid bilayer vesicle. The shape and size of RL were confirmed using electron microscopy and dynamic light scattering. A total of 96.3% of the RL had a uniform particle size of 110 nm diameter with a spherical shape (Figure S1). We then evaluated the cytotoxicity of RL on CRFK cells using a water-soluble tetrazolium salts (WST) assay with serial dilution of each compound. The 50% cytotoxic concentration (CC_50_) of RL was 3.53 µM, which was similar to R (3.90 µM) (R^2^ > 0.95, Figure 1A). Next, we determined the inhibitory effects of RL against FCoV in the cell culture at a concentration below the CC_50_. CRFK cells were pretreated with various concentrations of RL or R and then infected with FCoV (0.008 MOI) in the presence of RL or R. After 72 h post-infection, viral RNA synthesis, N protein expression, and virus titer were measured. As shown in Figure 1B–D, treatment with both RL and R significantly inhibited FCoV replication in a dose-dependent manner. All tested concentrations showed statistically significant differences compared to the mock test group. The effective concentrations inhibiting 50% viral replication (EC_50_) of RL and R were 1.40 µM and 1.60 µM, respectively (R^2^ > 0.95). Interestingly, RL showed a significantly enhanced antiviral effect (*p* < 0.05) compared to R in terms of viral RNA, protein, and titration, suggesting that loading R onto a liposome could increase its potency (Figure S2). To ensure that the observed antiviral effect of RL was not due to the liposome carrier, we tested the equivalent concentration of the liposome (L) and found that L treatment did not show significant differences from the mock experiment (Figure 1D and Figure S2), further supporting that the antiviral effect of RL is attributed to R.

### 3.2. RL Decreased PKC Delta Phosphorylation Induced by FCoV Infection at an Early Stage of Infection

To investigate whether RL inhibits FCoV replication through the PKCδ-dependent pathway, we tracked the phosphorylation of overtime after FCoV infection with or without RL. CRFK cells were infected with a mixture of FCoV (MOI of 1) and RL (2.5 μM), and the cell lysates were collected at 15, 30, 45, and 60 min after infection for Western blotting to measure total PKCδ, phosphorylated PKCδ (PKCδ-S645), and β-actin. 

**Figure 2:**
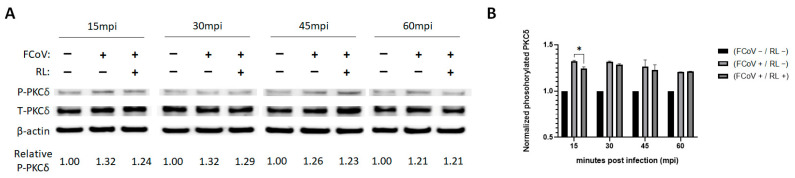
RL slightly reduced FCoV-induced PKCδ phosphorylation in CRFK cells at an early stage of infection. Pre-treated (2.5 μM of RL for 2 h) confluent CRFK cells were infected with FCoV (MOI = 1) and RL, and then cell lysates were collected 15, 30, 45, and 60 min post-infection. (**A**) Cell lysates were subjected to SDS-PAGE for Western blotting. The phosphorylated (P-PKCδ) and total PKCδ (T-PKCδ) were detected using rabbit anti-PKCδ-S645 and rabbit anti-PKCδ antibody, respectively. β-actin was employed as an internal control. Bands were quantified using Image J software. Values of P-PKCδ were normalized to those of T-PKCδ. The relative P-PKCδ was calculated as a fold change of the normalized values to the negative control (NC, FCoV−/RL−) of each time point. (**B**) The measured signals were plotted on a graph. Statistical differences were analyzed through two-way ANOVA and Tukey’s multiple comparison test, with the symbol (*) indicating *p* < 0.05 (please find the WB full membrane in Figure S3).

The levels of phosphorylated PCKδ in the FCoV-infected groups (FCoV+) were significantly increased compared to those of the negative control group (FCoV−/RL−) at all time points (1.21~1.32, *p* < 0.0001, not indicated), suggesting that FCoV infection induced the phosphorylation of PCKδ (Figure 2A,B). The RL-treated group exhibited a slightly decreasing trend in the average values of the phosphorylated PKCδ compared to the positive control group (FCoV+/RL−) at 15, 30, and 45 min after infection (Figure 2A,B). Statistical analysis showed that the RL treatment significantly reduced the phosphorylated PKCδ 15 min after infection (FCoV+/RL−, 1.32 ± 0.01 vs. FCoV+/RL+, 1.24 ± 0.02, *p* < 0.05) (Figure 2B). There were no significant differences in phosphorylated PKCδ 30, 45, or 60 min after infection. These results indicate that FCoV infection in CRFK cells induces PKCδ phosphorylation and RL treatment might decrease the phosphorylation at an early stage of infection.

### 3.3. RL Inhibited FCoV Replication at Both Early and Late Stages of Infection

**Figure 3:**
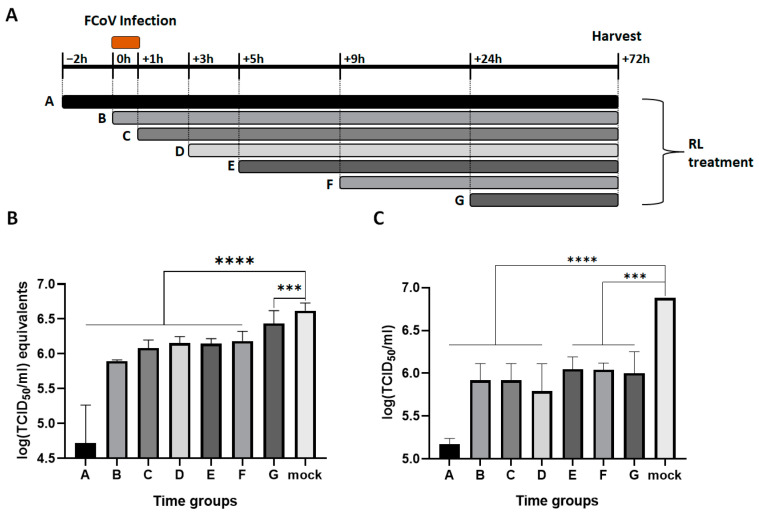
RL exhibits inhibitory effects in at least two different stages of FCoV replication. Confluent CRFK cells were infected with FCoV (MOI = 0.008) from +0 to +1 h. RL (2.5 μM) was added to the media at an indicated time point and maintained for +72 h. (**A**) Schematic diagram of the time of addition assay. (**B**) Cell lysates were subjected to extracting viral RNA for RT-qPCR. The Cq values were converted into log (TCID50/mL) equivalents with the standard curve. (**C**) The production of infectious viruses was quantified and presented as a log (TCID50/mL). Statistical differences were analyzed using one-way ANOVA and Dunnett’s multiple comparison tests. The symbols (****) and (***) indicate *p* < 0.0001 and *p* < 0.0005, respectively.

To investigate which stage of FCoV replication was inhibited by RL, a time of drug addition assay was conducted. The experimental design is presented in Figure 3A. During the period from −2 to +72 h, RL was added to the media at the indicated time points, and FCoV was inoculated from 0 to +1 h. After +72 h, each cell lysate was collected, and the viral RNA and infectious particle titers were measured. As shown in Figure 3B,C, RL treatment showed significant inhibition of both viral RNA and infectious virus titer for all time groups compared to the mock groups (*p*< 0.0001 or *p* < 0.0005). RL pre-treatment at −2 (group A) showed the highest reduction in viral RNA (4.7 ± 0.5 log (TCID_50_/mL) equivalent) and titer (5.2 ± 0.1 log (TCID_50_/mL)) among all groups, suggesting that RL affects an early stage of FCoV infection. RL treatment at +0, +1, +3, +5, and +9 (Group B, C, D, E, and F, respectively) resulted in the intermediate inhibition of viral replication. Interestingly, RL treatment at +24 h (group G) significantly decreased the viral titer but only slightly decreased the viral RNA. These intermediate inhibitions indicate that RL also affects the middle and late stages of FCoV infection, which may be associated with disrupting viral protein translation, capsid assembly, virus release, or virus spread. 

### 3.4. RL Decreased FCoV-Mediated Syncytia in Cell Cultures

**Figure 4:**
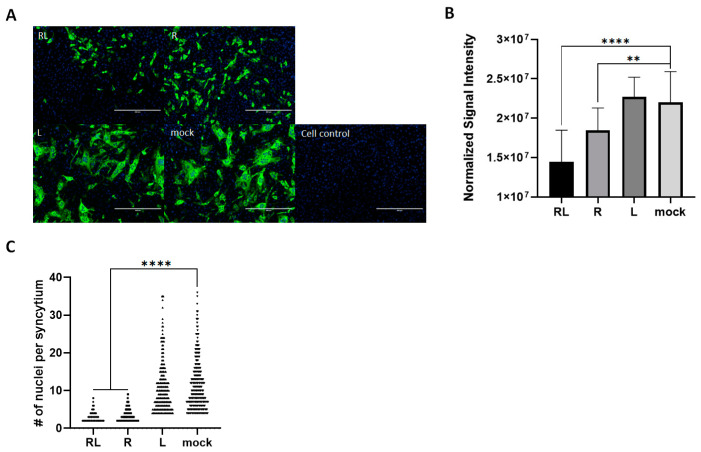
Rottlerin decreased the syncytial formation of FCoV. (**A**) Confluent CRFK cells were infected with FCoV (MOI = 0.008) and 2.5 μM of R, RL, or L for 24 h. Then, the cells were fixed, and the FCoV N protein was stained. The stained cells were observed under a fluorescence microscope (green = FCoV N protein, blue = nuclei, white scale bar = 400 μm). (**B**) The captured images were quantified and the intensity of the green fluorescence signal per cell was plotted. (**C**) The number of nuclei per syncytium (NPS) was quantified. The symbols (****) and (**) indicate *p* < 0.0001 and *p*< 0.005, respectively.

To explore the effect of RL on the FCoV growth pattern in the CRFK cells, we utilized an immunofluorescence assay to observe virus-infected cells in the presence or absence of RL. As expected, treatment with both RL and R resulted in a decrease in the number and intensity of signals compared to the mock and liposome controls. This is consistent with the titration and Western blot results (Figure 1), confirming that RL inhibited FCoV replication (Figure 4A). In the quantification analysis, both RL (1.4 × 10^7^ ± 4.0^6^) and R (1.8 × 10^7^ ± 2.8 × 10^6^) showed significantly lower fluorescence intensity than the mock (2.2 × 10^7^ ± 3.9 × 10^6^) or liposome (2.3 × 10^7^ ± 2.5 × 10^6^) groups (Figure 4A). Surprisingly, RL- and R-treated cells showed fewer syncytia formations than the mock and liposome-treated cells. To confirm our observation, we counted the average number of nuclei per syncytium, which is an indication of cell–cell fusion formation [51]. The average numbers of nuclei per syncytium of both RL- (3.37) and R (2.92)-treated cells were significantly lower than those of the mock (11.29) and liposome (10.58) cells (Figure 4B). These results indicate that RL might interfere with syncytia formation by FCoV at a late stage of infection. 

### 3.5. RL Inhibits FCoV Endocytosis

**Figure 5:**
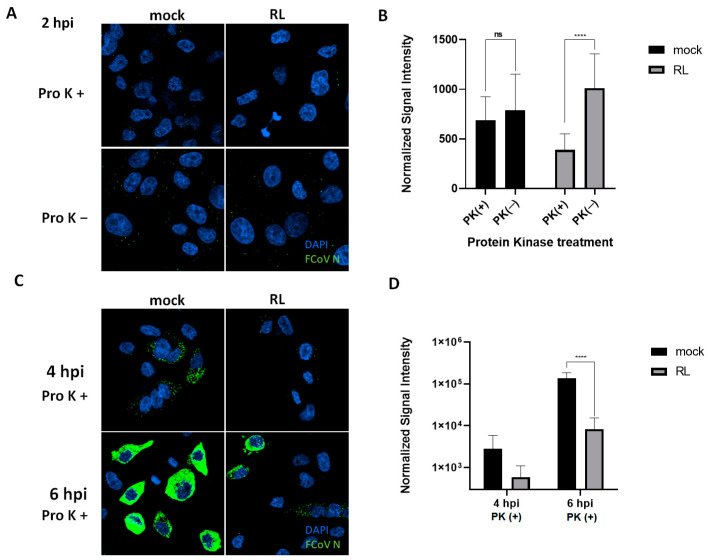
RL inhibited the internalization (or endosomal exit) of FCoV. (**A**,**B**) After treating confluent CRFK cells with RL (2.5 μM) for 2 h, a mixture of RL (2.5 μM) and FCoV (MOI = 0.008) was inoculated for 1 h and replenished with fresh media containing RL. After 2 h post-infection, the cells were treated with proteinase K (Pro K+) or PBS (Pro K−) for 45 min, then fixed and stained. The cells were analyzed using confocal microscopy (green = FCoV N, blue = nuclei). The intensity of the green signal per cell was quantified from the images using Image J and plotted. (**C**,**D**) As in panel (**A**), except with incubation for 4 and 6 h. The cells were proteinase-K-treated for 45 min. The symbols (ns) and (****) indicate *p* > 0.05 and *p*< 0.0001, respectively.

Since pre-treatment of RL showed the most significant reduction in FCoV replication, we hypothesized that RL treatment could disturb the entry process of FCoV. To test this hypothesis, we observed the early localization of FCoV in CRFK cells after treatment of RL or mock (DMSO) with or without proteinase K (proK). The proK treatment was capable of removing outside viruses from the plasma membrane, thereby enabling us to observe only endocytosed virus particles (Figure 5). Two hours post-infection, the mock group showed no difference in fluorescence signal intensity, with or without proK treatment. Strikingly, RL treatment significantly reduced the fluorescence signal intensity after proK treatment, suggesting that RL inhibits the endocytosis of FCoV (Figure 5A,B). At 4 and 6 h post-infection, RL treatment resulted in a significant reduction in fluorescence signal intensity compared to mock-treated cells, which is consistent with the previous results (Figure 5C,D). These results indicate that RL can inhibit the endocytosis of FCoV at an early stage of replication. 

## 4. Discussion

In this study, we have presented preliminary evidence showing that rottlerin liposome (RL) exhibits inhibitory activity against FCoV in CRFK cells. Rottlerin (R) has shown antiviral activity against many viruses, especially enveloped viruses, including chikungunya virus, dengue virus, human immunodeficiency virus-1 (HIV-1), human T-cell leukemia virus-1 (HTLV-1), la crosse virus, porcine reproductive and respiratory syndrome virus (PRRSV), rabies virus, rift valley fever virus, severe acute respiratory syndrome coronavirus 2 (SARS-CoV-2), and zika virus [6,10,11,12,13,16,52,53,54,55]. These results suggest that R ca be used as a broad-spectrum antiviral for emergency usage against potential future viral outbreaks. 

We showed that RL inhibited the replication of FCoV by interrupting its endocytosis (Figure 5). This effect seems to be related to the phosphorylation of PKCδ (Figure 2). These findings are corroborated by the time of drug addition assay, which revealed that the greatest inhibition of FCoV replication was achieved with pretreatment of RL (Figure 3). Notably, the inhibition of viral endocytosis by R has also been reported in other viruses, including PRRSV, zika virus, and puumala virus [6,12,56]. However, it is not yet clear how R can interfere with viral endocytosis. One possibility is that R interferes with viral endocytosis by regulating actin rearrangement in a PKCδ-dependent manner. In our previous study using PPRSV and RL, we observed that RL inhibited PRRSV endocytosis by reducing actin rearrangements, which is associated with cellular PKCδ phosphorylation (unpublished data). Another possible explanation is that R decreases ATP levels as a mitochondrial uncoupler, which acts in a PKCδ-independent manner. This decreased ATP level can inhibit the activities of several kinases as well as PKCδ [15]. Additionally, gene silencing of ATP1A1, a subunit of the multi-subunit Na^+^, K^+^-ATPase, resulted in a similar phenotype of R showing inhibition of the entry of mouse hepatitis virus [57]. In zika virus, R interferes with the virus endocytosis, but this effect was related to neither ATP nor the PKCδ pathway [6]. Therefore, it will be necessary to conduct a complementary experiment using PKCδ knock-down cells to confirm whether R inhibits FCoV endocytosis through PKCδ phosphorylation.

We have presented evidence demonstrating that RL also inhibits the late stage of FCoV replication. Treatment with R and RL on FCoV-infected cells resulted in a reduction in the number of syncytia formed (Figure 4). Furthermore, the time of addition assay showed that the viral titer (TCID_50_/mL) was significantly more inhibited than viral RNA (TCID_50_/mL equivalents) at +24 h (Figure 3). Based on these results, we infer that RL inhibits downstream stages of viral RNA synthesis. Two possible hypotheses may explain the effect of R on the late stage of FCoV replication and syncytia formation. Firstly, R may affect the process of protein synthesis from viral RNA as it has been shown to inhibit the translation of viral mRNA [7,58]. Second, R may directly or indirectly inhibit the function of the spike protein or fusion-related factors, similar to drugs such as EK1 and Abl kinase inhibitors [51,59]. Since mitochondria uncoupling can affect various cellular enzymes, it is also possible that R can inhibit multiple steps of FCoV replication, such as protein synthesis, viral capsid assembly, and trafficking [15]. To determine the underlying mechanism of R during the late stage of FCoV replication, we plan to conduct more detailed studies in the future by transfecting the mRNA-encoding FCoV S-protein together with RL or by treating the cells with an externally synthesized S protein with RL [60].

Taken together, these results suggest that R may inhibit FCoV replication via two distinct stages: an early stage involving endocytosis and a late stage involving viral protein synthesis or spread. Interestingly, R has been shown to exert antiviral effects against various viruses through PKCδ-dependent pathways (such as in HIV-1 and PRRSV) or PKCδ-independent pathways that reduce ATP levels (such as in rabies and zika viruses), suggesting that R may inhibit multiple stages of viral replication [10,12,16]. This finding is consistent with a recent Zika virus report stating that R disrupts both endocytosis and late-stage replication by interfering with virus maturation [6]. Nonetheless, the underlying mechanism by which R blocks multiple stages in some enveloped viruses requires further investigation.

We demonstrated the advantages of liposomes in enhancing the potency of R (Figure 1 and Figure 4). Liposomes offer additional advantages for the clinical use of R. First, liposome-based formulations can overcome challenges related to the solubility, bioavailability, pharmacological activity, stability, distribution, and sustained delivery of natural extracts [25,26]. Thus, liposomes are widely used in the field of natural extracts, such as R, with various combinations of lipids and sizes [61]. R is soluble in DMSO, ethanol, and chloroform, but is not soluble in water [58]. Therefore, liposomes enable the administration of a high dose of R in an affordable volume, safely using PBS or distilled water as a vehicle. The RL we produced in this study could accommodate a maximum of 2.5 mg/mL of R, providing us with a sufficient drug concentration in a small volume for future in vivo studies. Second, RL could increase its uptake by macrophages, which could enhance its antiviral effect against FCoV. Liposomes with a size under 100 nm are known to facilitate their uptake by macrophages [62]. FCoV infection in macrophages causes the pathogenesis of FIP, which is characterized by the release of inflammatory factors from macrophages, which leads to vessel leakage and the formation of granulomatous lesions [31,63,64]. Thus, if RL can specifically target macrophages, it may not only increase the efficiency of R but also decrease its toxicity. Hence, it is worth investigating the application of liposomes to R or other anti-FCoV drugs and evaluating their positive effects through in vivo studies.

The 50% cytotoxic concentration (CC_50_) values of R and RL measured in CRFK cells were 3.90 and 3.53 μM, respectively, which are lower than those of other known small-molecule inhibitors (Figure 1). The CC_50_ values of other compounds effective against FCoV ranged from 5.99 μM to 515.7 μM in various cell models [22,42,43,65,66,67,68]. These results raise safety concerns, as evaluating the safety of chemical substances during their development into pharmaceuticals is crucial [30]. However, a direct comparison of their CC_50_ values among different studies might be unreasonable due to differences in the cell models used. For example, when R was used in other studies with A549 or Vero cells, the CC_50_ values were 47.78 μM and 11.24 μM, respectively [6,53]. In a previous porcine study conducted by our group, a dose of 1 mg/pig did not cause any observed adverse effects [12]. However, the previous study was a single-dose administration test using pigs, and there are not enough data to prove the safety of its application to cats. Therefore, it is essential to conduct in vivo toxicity studies that meet the standards of clinical trials before applying R to cats, and we are planning subsequent studies on the toxicity of R and RL.

## 5. Conclusions

In this study, we have demonstrated that Rottlerin-Liposome (rottlerin encapsulated in liposomes) efficiently inhibits FCoV replication in cell cultures by reducing FCoV endocytosis. We have also observed that RL reduces both fluorescent signals for FCoV N protein and the formation of syncytia in FCoV-infected cells. Additionally, we have found that encapsulating rottlerin in liposomes enhances its inhibitory effect compared to using rottlerin alone. These results suggest that RL has the potential to be further investigated for its antiviral activity.

## Data Availability

Data are available from the corresponding author upon reasonable request.

## Supplementary Materials

The following supporting information can be downloaded at https://www.mdpi.com/article/10.3390/vetsci10060380/s1: Figure S1: RLs have a spherical structure and a uniform size. (A) The shapes of the RLs were observed using a transmission electron microscope (TEM). The scale bar (white) indicates 200 nm. (B) The size distribution of the RLs was measured using a dynamic light scattering (DLS) system. The graph shows the intensity distribution according to the radius of the particles, and the table below shows the radius and intensity values of each peak.; Figure S2: The anti FCoV effect was due to R, but not liposomes. After pre-treating the cells with 2.5 μM of R, RL, or liposome (L) (−2hpi), FCoV (0.008 MOI) was inoculated to the CRFK cells with the agents. Cell lysates were collected after three days and tested via titration using the 10-fold serial dilution method. Infectious virus titer was measured using Reed–Mench. The symbols (****) and (*) indicate *p* < 0.0001 and *p* < 0.05, respectively; Figure S3: WB full membrane for Figure 1D and Figure 2.
