# Supplementary material for: Rottlerin-Liposome Inhibits the Endocytosis of Feline Coronavirus Infection
Source: Vet Sci. 2023 May 30;10(6):380. doi: 10.3390/vetsci10060380 (PMC10302841; doi:10.3390/vetsci10060380)
Supplement: Supplementary file 1 [file vetsci-10-00380-s001.zip › figure_s3.pdf]

Western blot original images

Fig1D.

Anti FIPV-N antibody

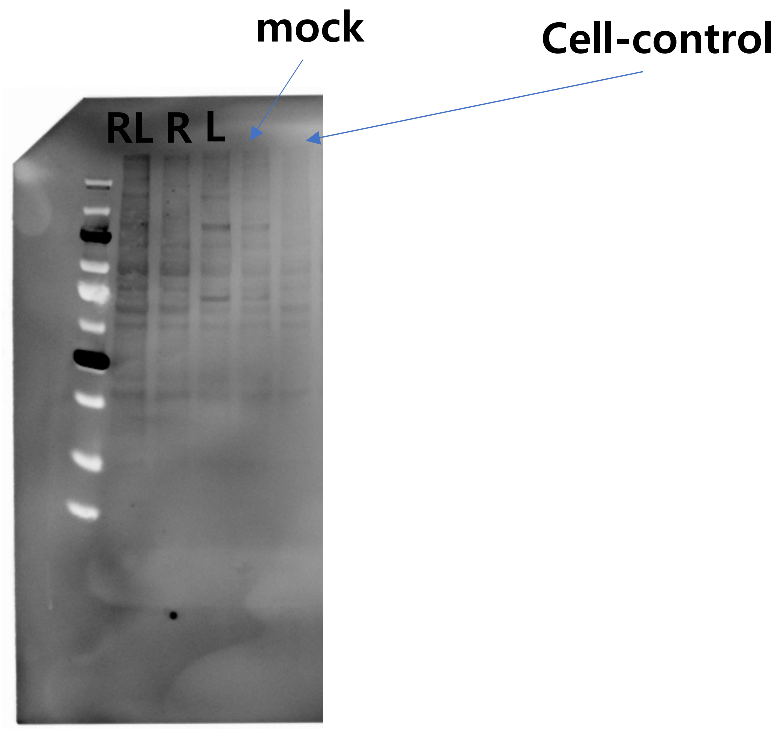

Anti β-actin antibody

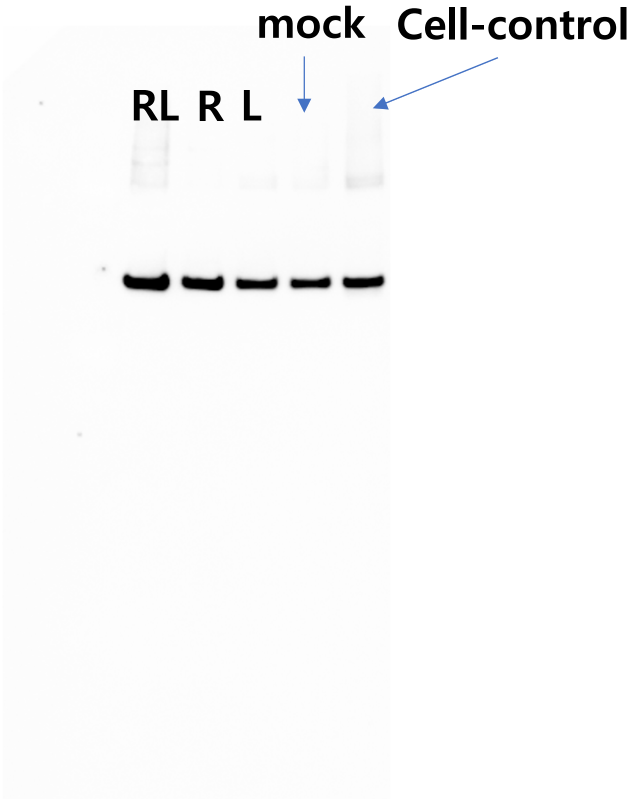

Replication #1

Fig2.

1	2	3	4	5	6	7	8	9	10	11	12	marker
15mpi (FIPV - / RL -)	15mpi (FIPV + / RL -)	15mpi (FIPV +/RL +)	30mpi (FIPV - / RL -)	30mpi (FIPV + / RL -)	30mpi (FIPV +/RL +)	45mpi (FIPV - / RL -)	45mpi (FIPV + / RL -)	45mpi (FIPV +/RL +)	60mpi (FIPV - / RL -)	60mpi (FIPV + / RL -)	60mpi (FIPV +/RL +)	Invitrogen™ iBright™ Prestained Protein Ladder LC5615

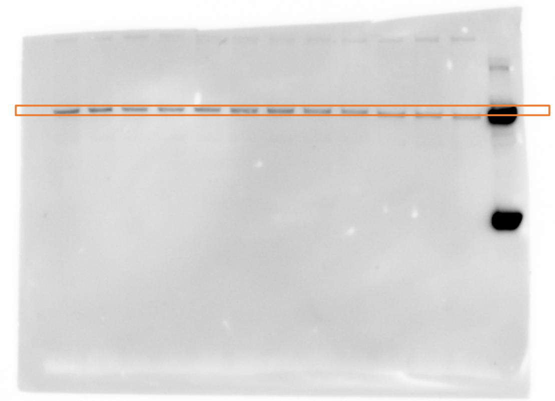

Anti PKCδ-S645 antibody (phosphorylated PKCδ)

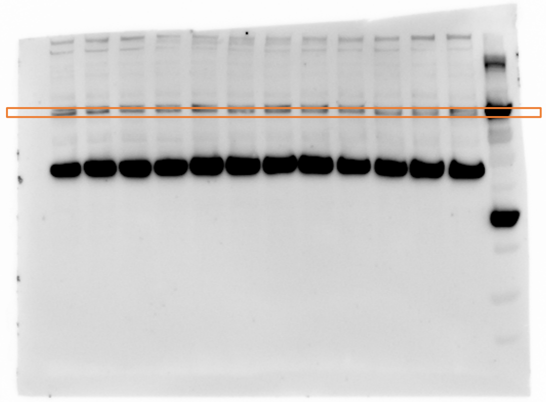

Anti PKCδ antibody (total PKCδ)

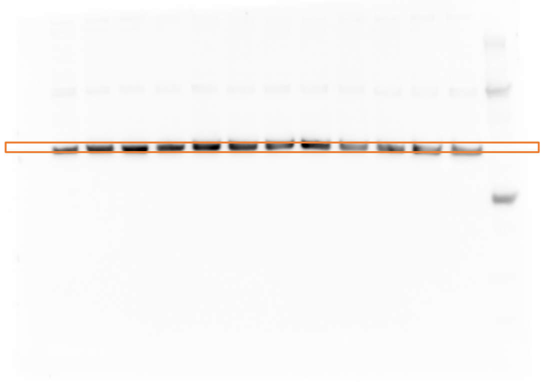

Anti β-actin antibody

Replication #2, #3

Fig2.

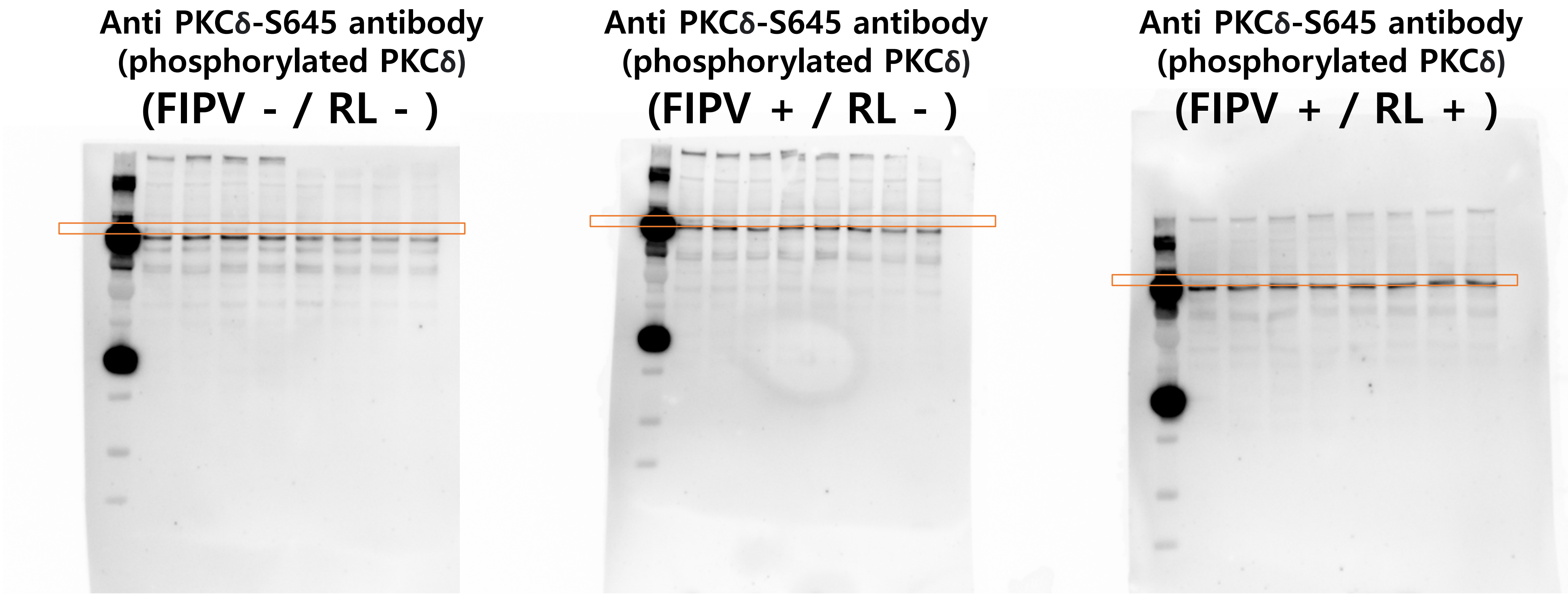

marker	1	2	3	4	5	6	7	8
Invitrogen™ iBright™ Prestained Protein Ladder LC5615	15mpi Replication #2	15mpi Replication #3	30mpi Replication #2	30mpi Replication #3	45mpi Replication #2	45mpi Replication #3	60mpi Replication #2	60mpi Replication #2

Replication #2, #3

Fig2.

Anti PKCδ antibody (total PKCδ)

(FIPV - / RL -)

Anti PKCδ antibody (total PKCδ)

(FIPV + / RL -)

Anti PKCδ antibody (total PKCδ)

(FIPV + / RL +)

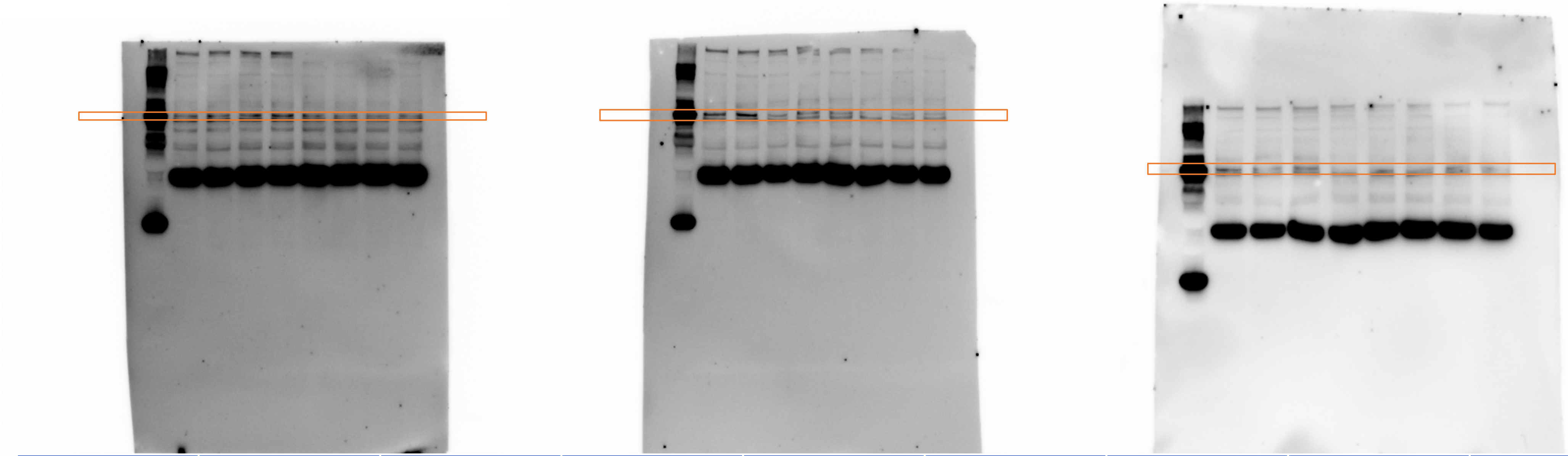

marker	1	2	3	4	5	6	7	8
Invitrogen™ iBright™ Prestained Protein Ladder LC5615	15mpi Replication #2	15mpi Replication #3	30mpi Replication #2	30mpi Replication #3	45mpi Replication #2	45mpi Replication #3	60mpi Replication #2	60mpi Replication #2

Replication #2, #3

Fig2.

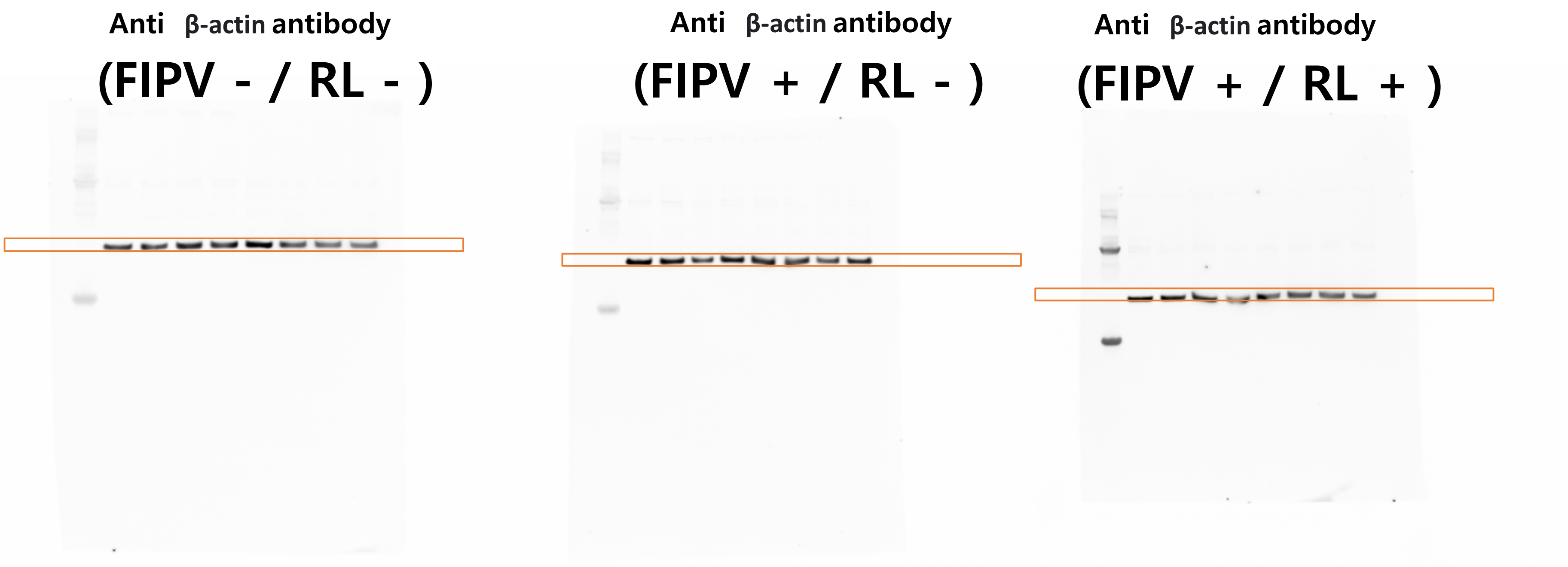

marker	1	2	3	4	5	6	7	8
Invitrogen™ iBright™ Prestained Protein Ladder LC5615	15mpi Replication #2	15mpi Replication #3	30mpi Replication #2	30mpi Replication #3	45mpi Replication #2	45mpi Replication #3	60mpi Replication #2	60mpi Replication #2

Replication #4

Fig2.

1	2	3	4	5	6	7	8	9	10	11	12	marker
15mpi (FIPV - / RL -)	15mpi (FIPV + / RL -)	15mpi (FIPV +/RL +)	30mpi (FIPV - / RL -)	30mpi (FIPV + / RL -)	30mpi (FIPV +/RL +)	45mpi (FIPV - / RL -)	45mpi (FIPV + / RL -)	45mpi (FIPV +/RL +)	60mpi (FIPV - / RL -)	60mpi (FIPV + / RL -)	60mpi (FIPV +/RL +)	Invitrogen™ iBright™ Prestained Protein Ladder LC5615

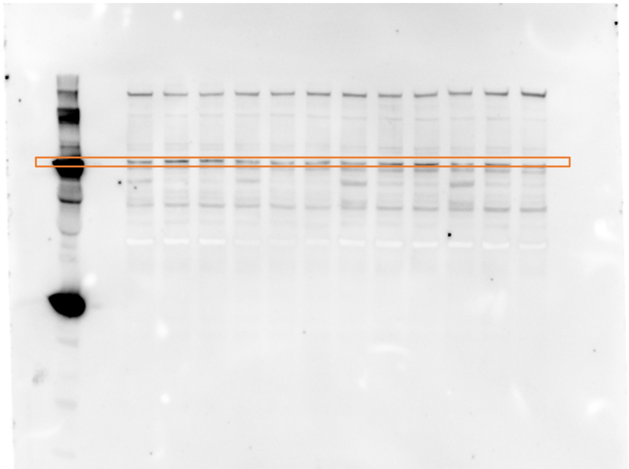

Anti PKCδ-S645 antibody (phosphorylated PKCδ)

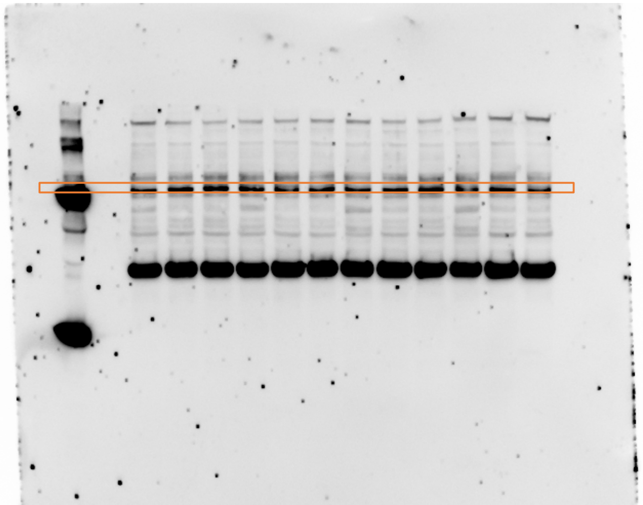

Anti PKCδ antibody (total PKCδ)

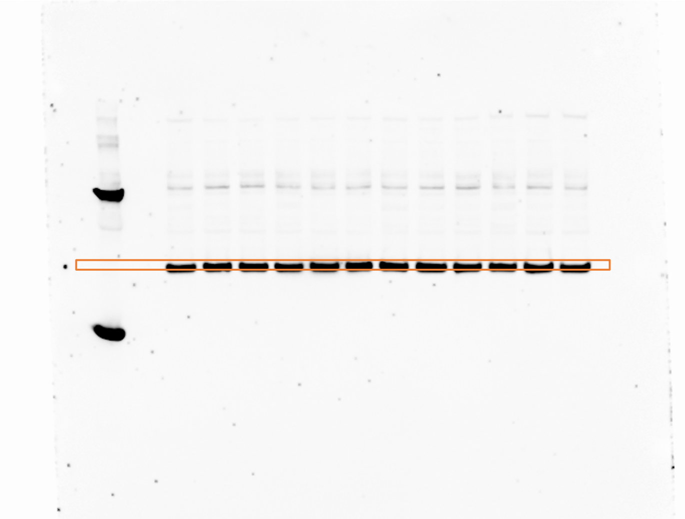

Anti β-actin antibody
