# Supplementary figures and images for: Rottlerin-Liposome Inhibits the Endocytosis of Feline Coronavirus Infection
Source: Vet Sci. 2023 May 30;10(6):380. doi: 10.3390/vetsci10060380 (PMC10302841; doi:10.3390/vetsci10060380)

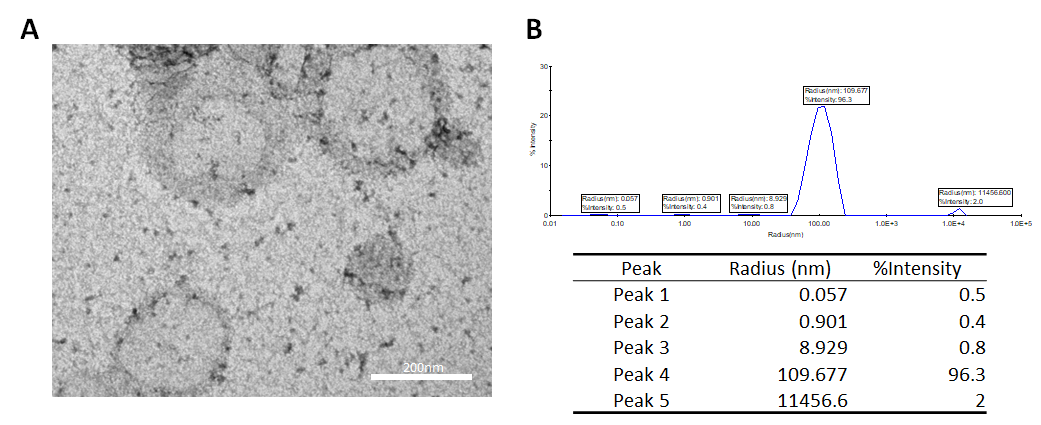

Supplement: Supplementary file 1 [file vetsci-10-00380-s001.zip › figure_s1.tif]

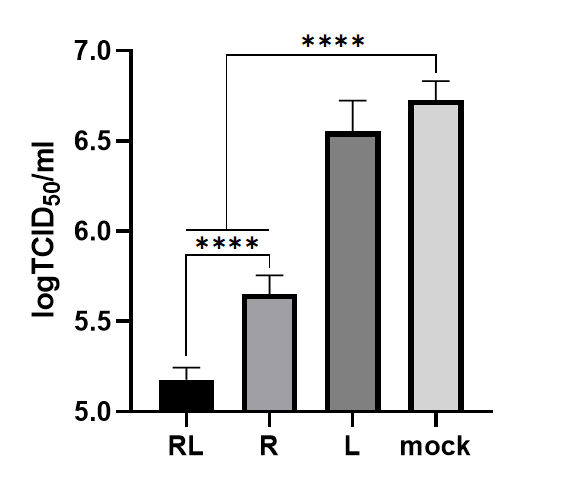

Supplement: Supplementary file 1 [file vetsci-10-00380-s001.zip › figure_s2.tif]
